# Seoul Virus Infection in Humans, France, 2014–2016

**DOI:** 10.3201/eid2306.160927

**Published:** 2017-06

**Authors:** Jean-Marc Reynes, Damien Carli, Jean-Baptiste Bour, Samir Boudjeltia, Anny Dewilde, Guillaume Gerbier, Timothée Nussbaumer, Véronique Jacomo, Marie-Pierre Rapt, Pierre E. Rollin, Alexandra Septfons

**Affiliations:** Institut Pasteur, Lyon, France (J.-M. Reynes, D. Carli);; Centre Hospitalier Universitaire Dijon-Bourgogne, Dijon, France (J.-B. Bour, S. Boudjeltia);; Centre Hospitalier Universitaire Lille, Lille, France (A. Dewilde);; Direction Départementale de la Cohésion Sociale et de la Protection des Populations, Colmar, France (G. Gerbier);; Hôpitaux Civils de Colmar, Colmar (T. Nussbaumer); Biomnis, Lyon (V. Jacomo);; Centre Hospitalier, Bar-le-Duc, France (M.-P. Rapt);; Centers for Disease Control and Prevention, Atlanta, Georgia, USA (P.E. Rollin);; Santé Publique France, Saint-Maurice, France (A. Septfons)

**Keywords:** Seoul virus, viruses, hantavirus, infection, humans, zoonoses, brown rats, Rattus norvegicus, France

## Abstract

We report detection of Seoul virus in 3 patients in France over a 2-year period. These patients accounted for 3 of the 4 Seoul virus infections among 434 hantavirus infections (1.7%) reported during this time. More attention should be given to this virus in Europe where surveillance has been focused mostly on Puumala and Dobrava-Belgrade hantaviruses.

Seoul virus (SEOV), a hantavirus and the etiologic agent of a mild-to-moderate hemorrhagic fever with a renal syndrome, is associated worldwide with brown rats (*Rattus norvegicus*), a commensal rodent that is found in all human-inhabited locations (*1*). In Russia, South Korea, and China, a wide range (few tens to few thousands) of human infections with SEOV are reported annually (with potential serologic cross-reactivity with co-circulating Hantaan virus); otherwise, only a few countries have reported rare sporadic cases, mostly serologically confirmed (*1**–**4*). Four human cases were recently described in the United Kingdom and France (*4**–**6*). We report 3 patients in France, whose SEOV infections were confirmed by using molecular techniques, and identified through systematic surveillance within a 24-month period.

## The Study

Case-patient 1 was a 27-year-old man from Dijon, France, who was hospitalized 3 days after onset of symptoms during February 2014. His clinical and biologic characteristics were fever, mild renal syndrome, thrombocytopenia, and increased levels of liver enzymes (Table 1). Hantavirus IgM and IgG were detected in an admission serum sample by using commercial ELISAs at a private clinical laboratory. Presence of IgM was confirmed at the French National Reference Center for Hantavirus (NRC; Lyon, France) by using reference ELISA and indirect fluorescent antibody assays (*5*) (Table 2).

**Table 1:** Characteristics of 3 patients infected with Seoul virus, France, 2014–2016*

Characteristic†	Patient 1	Patient 2	Patient 3
Signs and symptoms			
Fever	Yes	Yes	Yes
Weakness	Yes	No	Yes
Headache	Yes	Yes	Yes
Muscle or join pain	No	No	Yes
Chest pain	No	No	Yes
Abdominal pain	No	No	Yes
Nausea	Yes	No	Yes
Vomiting	Yes	No	Yes
Anorexia	No	No	Yes
Cough	Yes	No	No
Dyspnea	No	No	Yes
Anuria	No	No	Yes
Confusion	Yes	No	Yes
Laboratory results (reference range or value)			
Leukocytes, × 10^9^ cells/L (4.0–10.0 × 10^9^ cells/L)	Reference	Reference e	D6: 19.4
Platelets, × 10^9^/L (150–450 × 10^9^/L)	D4: 52	D4: 59	D4: 21
Hemoglobin, g/dL (13.0–17.0 g/dL)	D6: 11.9	Reference e	D13: 9.8
C-reactive protein, mg/L (<3.2 mg/L)	D3: 82.0	D4: 26.0	D4: 155.0
LDH, IU/L (120–228 IU/L)	D3: 999‡	NA	D4: 876
AST, IU/L (15-37 IU/L)	D3: 440	D5: 183	D4: 439
ALT, IU/L (21–72 IU/L)	D9: 278	D5: 210	D4: 390
GGT, IU/L (15–85 IU/L)	Reference	D5: 87	D9: 722
Creatinine, mg/L (6–11 mg/L)	D3: 16.4	Reference	D8: 75.0
Microscopic hematuria	NA	No	Yes
Proteinuria, g/24 h (<0.3 g/24 h)	NA	NA	D8: 3.82

*ALT, alanine aminotransferase; AST, aspartate aminotransferase; D, day after symptom onset; GGT γ-glutamyl transferase; LDH, lactate dehydrogenase; NA, not available;  †Dysphagia, diarrhea, jaundice, sore throat, rash, bleeding manifestation, and loss of consciousness were not observed for these patients.  ‡Patient 1 also had posttraumatic rhabdomyolysis: myoglobin level 869.5 μg/mL at D3 (reference range 16.0–116.0 µg/mL) and creatine kinase level 9,444 μg/mL at D5 (reference range 39–308 μg/mL).

**Table 2:** Serologic results for hantaviruses for 3 patients with virologically confirmed infections with Seoul virus, France, 2014–2016*

Patient no.	Day of sampling†	First-line diagnostic test, commercial ELISA (index value)		Diagnostic confirmation at NRC								Additional test at NRC‡	
				ELISA						IFA			
				IgM			IgG			Ig		IgM
		IgM	IgG	SEOV	PUUV		SEOV	PUUV		SEOV	PUUV	PUUV
1	3	+§ (4.3)	+§ (1.9)	+	–		–	–		–	–	–
2	5	+¶ (7.9)	+¶ (>200)	+	–		E	–		+	E	–
	29	ND	ND	+	E		+	E		+	–	ND
3	4	+§ (11.8)	+§ (9.8)	+	+		E	–		+	–	–
	80	ND	ND	+	–		+	+		+	+	ND

*E, equivocal; IFA, indirect fluorescent antibody; ND, not done; NRC, National Reference Center for Hantavirus (Lyon, France); PUUV, Puumala virus, SEOV, Seoul virus; +, positive; –, negative.  †No. days after symptom onset. ‡Reascan Rapid Test (Reagena, Toivala, Finland). §Hantavirus IgG DxSelect and Hantavirus IgM DxSelect (Focus Diagnostics, Cypress, CA, USA).  ¶Hantavirus Pool 1 Eurasia IgG and IgM (Euroimmun AG, Lübeck, Germany).

**Figure 1:**
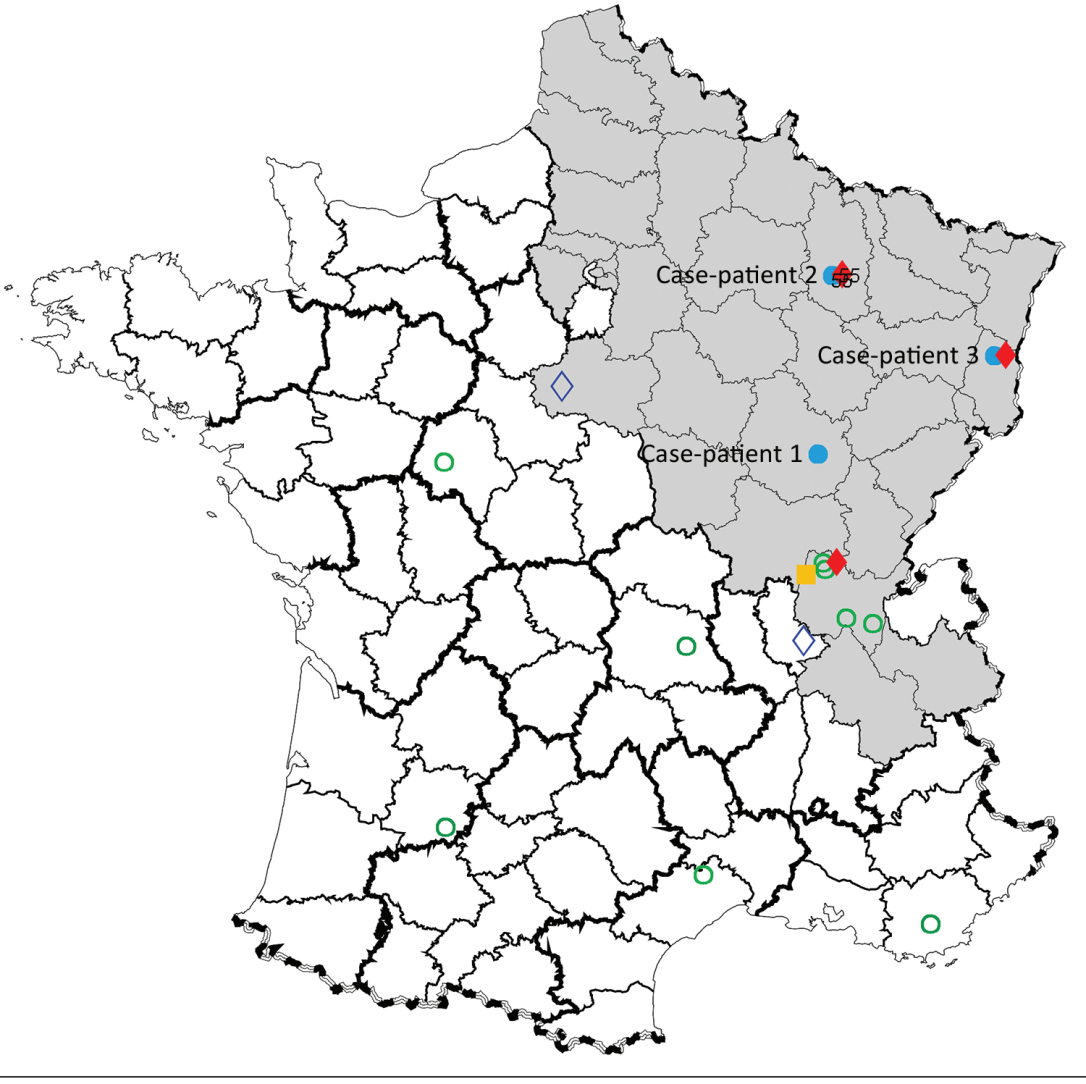
Geographic distribution of Seoul virus (SEOV) infections among human and rats, France 2016. Gray shading area indicates area of France to which Puumala virus is endemic. Open green circles indicate SEOV serologically confirmed human infections with SEOV reported by Ragnaud et al. (*10*), Le Guenno (*11*), and Bour et al. (*6*); solid blue circles indicate virologically confirmed human infections with SEOV reported in this study (patients 1, 2, and 3); solid yellow square indicates virologically confirmed human infection with SEOV reported by Macé al. (*5*); solid red diamonds indicate virologically confirmed SEOV infections in brown rats reported in this study; and open blue diamonds indicate virologically confirmed SEOV infections in brown rats reported by Heyman et al. (*12*) and Dupinay et al. (*13*).

Virus was detected by using molecular techniques as described (*5*). A partial SEOV small RNA sequence (GenBank accession no. KX064269) was isolated from an admission sample by conducting a BLAST (https://blast.ncbi.nlm.nih.gov/Blast.cgi) search. Exposure to the virus was suspected to have occurred during building restoration work (Figure 1).

Case-patient 2 was a 22-year-old man from Erize-Saint-Dizier, France (Figure 1), who was hospitalized 4 days after onset of symptoms during September 2014. His clinical and biologic characteristics were fever, thrombocytopenia, and liver function disorders (Table 1). Hantavirus IgM and IgG were detected in an admission serum sample by using commercial ELISAs at a public hospital clinical laboratory. Results were confirmed at the NRC (Table 2).

Virus was detected by using molecular techniques, and a partial SEOV small RNA sequence was obtained from a serum sample, as shown by a BLAST search. 

The pet rat (*Rattus norvegicus*) of case-patient 2, bought ≈1 month before onset of symptoms, was considered the presumptive source of the virus. The animal was euthanized after consent of the patient was obtained. An identical partial SEOV small RNA sequence was obtained from the liver of the animal.

Case-patient 3 was a 32-year-old man from Turckheim, France (Figure 1), who was hospitalized 4 days after onset of symptoms during January 2016. His clinical characteristics were severe fever, thrombocytopenia, liver disorders, myopericarditis, and renal syndrome that require hemodialysis (Table 1). Hantavirus IgM and IgG were detected 6 days after symptom onset by using commercial assays in a private clinical laboratory. Results were confirmed at the NRC (Table 2). 

Virus was detected by using molecular techniques reported by Klempa et al. (*7*). A partial SEOV large RNA sequence (GenBank accession no. KX064268) was obtained from an admission serum sample, as demonstrated by a BLAST search.

This case-patient raised brown rats as a food source for his snakes and routinely captured and killed wild brown rats that invaded his hen house. His breeding unit was the likely source of human infection. Organs from 10 rats sampled at this unit were positive for SEOV by nested reverse transcription PCR (*7*). A partial large RNA sequence obtained from a rat was identical to that obtained from the case-patient. Only 1 wild rat caught near the hen house was sampled; results were negative for SEOV.

**Figure 2:**
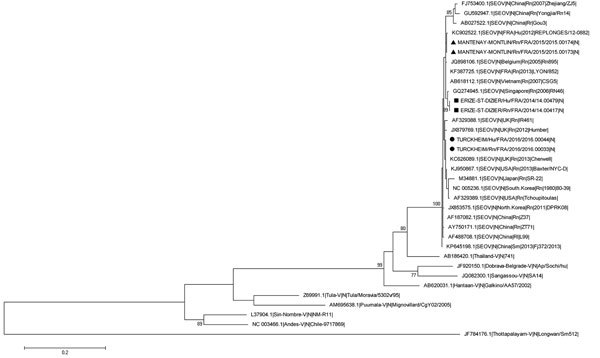
Phylogenetic tree based on the complete small RNA nucleotide coding sequences of Seoul virus (SEOV) strains isolated from 3 patients and rodents in contact with the patients infected with SEOV, France 2014–2016, and representative strains of SEOV and other hantavirus species. Triangles indicate sequences of strains detected in wild brown rats (this study) associated with a serologically confirmed human infection reported by Bour A et al. (*6*); squares indicate sequences of strains detected in case-patient 2 and in his pet brown rat; and circles indicate sequences of strains detected in case-patient 3 and 1 of his farmed brown rats. Bootstrap percentages >70% (from 500 resamplings) are indicated at each node; GenBank accession numbers are indicated for reference strains.Scale bar indicates nucleotide substitutions per site.

We obtained complete small RNA coding domain sequences (GenBank accession nos. KX064270–KX064275) from specimens from case-patients 1 and 2 (samples were not available for case-patient 1); specimens from the pet brown rat and raised brown rats suspected to be sources of infection for case-patients 2 and 3; and specimens from 2 wild brown rats suspected to sources of infection for a serologically confirmed human infection with SEOV detected in 2014 (*6*). Using MEGA version 5.1 (*8*) and a generalized time-reversible model with a gamma distribution and 5 rate categories (according to the best fit substitution model proposed), we performed phylogenetic analysis of the small RNA coding sequence. This analysis confirmed that all virus strains detected were SEOV (Figure 2).

## Conclusions

Four hantaviruses (Puumala [PUUV], SEOV, Tula, and Nova viruses), have been detected in France; the first 3 viruses were associated with humans, and most human infections were with PUUV (*9*). Human SEOV infection in Europe was initially reported in 2013 (*5*); however, human SEOV infections were previously suspected by serologic analysis in France (6 cases were reported during 1977–1996). SEOV was also detected in rodents from several areas in France (Figure 1) (*10**–**13*).

Detection of SEOV in 3 case-patients in our study and a recent report of a human infected with SEOV (*6*) within a 2-year period indicate that SEOV infections in humans are not uncommon in France. These infections accounted for 4 (1.7%) of 234 cases of hantavirus infection, mostly with PUUV, detected serologically or virologically during the same period in France. Detection of such cases might be caused by improvements in detection of SEOV infections, rather than by emergence of SEOV infections (SEOV antigen in serologic assays and molecular detection have been used at NRC since 2012).

Some infections with SEOV are probably missed in France. Commercials kits are used for hantavirus serologic diagnosis by 15 public hospital or private clinical laboratories in France. Eleven of these hospitals used a POC Puumala IgM rapid test (Reagena, Toivala, Finland). Four other hospitals used IgM and IgG ELISA kits with mixtures of recombinant antigens, including those from SEOV or Hantaan virus strains.

Most (83.4%) diagnostic tests in 2014 were requested for patients in the area of France to which PUUV is endemic. Consequently, some SEOV infections might have been be missed because hantavirus infection is rarely suspected outside this area. Samples with positive results for SEOV are sent to NRC for diagnostic confirmation and surveillance purposes. The 3 case-patients we report initially showed positive results by ELISA at local laboratories but then showed negative results by a ReaScan Puumala IgM test (Reagena) at NRC (Table 2). Therefore, SEOV infections were probably not detected initially by the 11 local laboratories who used the PUUV IgM Rapid Test, which led to underestimation of SEOV infections in humans in France.

This negative result could have also occurred in other countries in Europe that used the same test. Consequently, use of a pan hantavirus serologic assay is preferred. Furthermore, PUUV was detected by molecular techniques for most PUUV-infected patients during the acute phase of the disease (*14*). Thus, although there are no similar data for SEOV, to avoid misdiagnosis, we suggest using molecular diagnostic tests to more specifically detect hantavirus infections.

Furthermore, epidemiologic investigations showed that several animal traders in Europe sold pet rats or food rats to case-patients 2 and 3. These investigations also identified weak traceability of rat batches, absence of health-monitoring data for rats, and no information for the 3 case-patients on zoonotic risks for infection. Awareness of rat owners, traceability, and health monitoring of these animals, as performed for laboratory rats (*15*), should be improved.
